# Application of Machine Learning to Predict Acute Kidney Disease in Patients With Sepsis Associated Acute Kidney Injury

**DOI:** 10.3389/fmed.2021.792974

**Published:** 2021-12-10

**Authors:** Jiawei He, Jin Lin, Meili Duan

**Affiliations:** Department of Critical Care Medicine, Beijing Friendship Hospital, Capital Medical University, Beijing, China

**Keywords:** intensive care unit, sepsis, acute kidney injury, acute kidney disease, machine learning

## Abstract

**Background:** Sepsis-associated acute kidney injury (AKI) is frequent in patients admitted to intensive care units (ICU) and may contribute to adverse short-term and long-term outcomes. Acute kidney disease (AKD) reflects the adverse events developing after AKI. We aimed to develop and validate machine learning models to predict the occurrence of AKD in patients with sepsis-associated AKI.

**Methods:** Using clinical data from patients with sepsis in the ICU at Beijing Friendship Hospital (BFH), we studied whether the following three machine learning models could predict the occurrence of AKD using demographic, laboratory, and other related variables: Recurrent Neural Network-Long Short-Term Memory (RNN-LSTM), decision trees, and logistic regression. In addition, we externally validated the results in the Medical Information Mart for Intensive Care III (MIMIC III) database. The outcome was the diagnosis of AKD when defined as AKI prolonged for 7–90 days according to Acute Disease Quality Initiative-16.

**Results:** In this study, 209 patients from BFH were included, with 55.5% of them diagnosed as having AKD. Furthermore, 509 patients were included from the MIMIC III database, of which 46.4% were diagnosed as having AKD. Applying machine learning could successfully achieve very high accuracy (RNN-LSTM AUROC = 1; decision trees AUROC = 0.954; logistic regression AUROC = 0.728), with RNN-LSTM showing the best results. Further analyses revealed that the change of non-renal Sequential Organ Failure Assessment (SOFA) score between the 1st day and 3rd day (Δnon-renal SOFA) is instrumental in predicting the occurrence of AKD.

**Conclusion:** Our results showed that machine learning, particularly RNN-LSTM, can accurately predict AKD occurrence. In addition, Δ SOFA_non−renal_ plays an important role in predicting the occurrence of AKD.

## Introduction

The prevalence of acute kidney injury (AKI) in patients admitted to intensive care units (ICU) is approximately 50%. Nearly half of all AKI cases are present with sepsis, which may further worsen the prognosis (1, 2). Previous studies have reported the mortality rate of ICU patients with septic AKI as 30–45%, with the survivors still associated with the increased risk of chronic kidney disease (CKD) and cardiovascular events (3).

Increased severity and higher duration of AKI are associated with poor prognosis. In line with several previous results, Kellum et al. reported poorer clinical outcomes in patients with AKI lasting longer than 7 days than in patients who had renal function recovered within 7 days (4). Similar results have been previously reported in other studies (5, 6). Furthermore, in patients who developed sepsis persistent AKI beyond 7 days was associated with adverse clinical outcomes (5, 6). Hence, Acute Disease Quality Initiative-16 (ADQI-16) workshop suggested defining acute kidney disease (AKD) as impaired kidney function lasting 7–90 days after AKI (7). Unlike AKI patients, whose renal function typically recovers within 7 days, AKD patients suffer from persistent renal impairment and often have poor clinical outcomes (8).

Recent studies have utilized machine learning techniques for predicting AKI. Using machine learning techniques such as logistic regression and extreme gradient boosting (XGBoost), Zhang et al. identified some important clinical factors associated with AKI such as age, urinary creatinine concentration, maximum blood urea nitrogen concentration, and albumin (9). Zimmerman et al. showed that comprehensive demographics and physiologic features can accurately predict max serum creatinine level during day 2 and day 3 and also predict new AKI onset by cross-validation on linear regression and multiple machine learning models (10). However, AKD prediction has not been reported.

The AKD phase is a time window for potentially initiating key interventions to alter the natural history of kidney disease (7), and thus, the early identification of patients at high risk of developing AKD is important. Previous studies have shown that age, hypertension, diabetes mellitus, the history of CKD, the severity of AKI, and the use of mechanical ventilators were associated with the onset of AKD (11–17), however, machine learning methods have been seldom used to predict the occurrence of AKD. This study was aimed at using longitudinal data to predict the occurrence of AKD.

## Materials and Methods

### Data Source and Participants

Patients were recruited from the intensive care unit of Beijing Friendship Hospital (BFH), between January 1, 2015 and December 21, 2020. We obtained electronic healthcare data from Medical Information Mart for Intensive Care III (MIMIC III) (18). The inclusion criteria were as follows: (1) age ≥ 18 years old; (2) AKI caused by sepsis. The exclusion criteria were as follows: (1) AKI duration <48 h; (2) length of survival time <7 days; (3) CKD stage 5 or end-stage kidney disease defined as estimated glomerular filtration rate <15 ml/min/1.73 m^2^; (4) patients with missing important data (e.g., data on demographics and variables for calculating traditional severity scores). The study was reported according to the recommendations of the Transparent Reporting of a multivariable prediction model for Individual Prognosis or Diagnosis (TRIPOD) statement (19).

### Data Extraction

We extracted the following data from BFH and the MIMIC III records upon admission to ICU (day 1): (1) demographic information; (2) ICU details, including vitals, laboratory data, mechanical ventilation requirement, and exposure to nephrotoxic drugs; (3) severity of illness was measured using Simplified Acute Physiology Score II (SAPS II), Acute Physiological Score III (APS III), and non-renal Sequential Organ Failure Assessment (SOFA) score. The data on non-renal SOFA, creatinine, and urine output were recorded daily until day 3. Delta non-renal SOFA, delta creatinine, and delta urine output was the difference between the value at day 3 and the admission value.

### Outcomes and Definitions

The occurrence of AKD was the primary outcome. AKD was defined as the presentation of at least KDIGO Stage 1 criteria for >7 days after an AKI-initiating event, which agrees with the diagnostic criteria proposed by ADQI-16 in 2017 (7). The definition of sepsis was based on the diagnostic criteria of the Third International Consensus Definitions for Sepsis and Septic Shock (Sepsis-3), including a suspected infection and a SOFA score of ≥2 (20). The Kidney Disease: Improving Global Outcomes (KDIGO) classification according to both serum creatinine (SCr) and urine output (UO) criteria were used to define AKI (21). CKD was defined according to the Clinical Practice Guideline for the Evaluation and Management of Chronic Kidney Disease (22).

### Sample Size

The sample size was defined as having at least 10 outcome events per variable per estimated parameter according to a previous study (23). Our sample and the number of AKD approached that determined by the calculated result.

### Statistical Analysis

Values were presented as total numbers (percentages) for categorical variables and the means ± SDs or medians (interquartile ranges) for continuous variables. Comparisons were made using the Student's *t*-test or rank-sum test for continuous variables, and the Chi-square test or Fisher's exact test for categorical variables, as appropriate. All statistical tests were two-sided, and *P*-values of <0.05 were considered statistically significant.

### Model Development and Validation

The included patients from BFH and MIMIC III comprised the training dataset and the validation dataset, respectively. We selected three models for comparison: Recurrent Neural Network-Long Short-Term Memory (RNN-LSTM), decision tree, and logistic regression. The discrimination performance of these models in the training dataset and the validation dataset was evaluated by area under the receiver operating characteristic (AUROC).

### Recurrent Neural Network-Long Short-Term Memory

The RNN has been widely used to handle the longitudinal variables, LSTM is one type of RNN (24, 25). It can effectively process a large amount of sequential data. It comprises several modules, which can store the processed data from the previous stage. Unlike ordinary RNN, classic LSTM comprises several modules called cells. Data can be transferred from the previous cell to the next cell, including input gate, forget gate, and output gate. All data are added to the input gate, and the output gate displays the final data result. Unlike ordinary RNN which can have only one memory stacking method, LSTM can control the transmission state through the gating state, remember important information and forget unimportant information. The forget gate can enhance the ability of LSTM to process data and avoid the problem of data dependence.

### Decision Tree

Decision tree/random forest can predict the classification (AKD or non-AKD) from the data, which can display the decision result more clearly (26). We can use the decision tree to interpret the prediction results. The process from the root to the leaf of the tree shows the prediction classification, according to the algorithm of the decision tree. Each step of the decision tree involves checking a piece of data. If the predictor satisfied a certain condition, it would follow the upper branch to indicate type 0, predicting that AKD will occur. Otherwise, it would follow the lower branch to indicate type 1, predicting that AKD will not occur. The decision trees were trained to create a model that could factor in multiple input variables and predict the value of the target variable. The division of the tree continues until the node contains the minimum number of training examples or reaches the maximum tree depth. The complexity parameter is used to indicate the prediction performance, which depends on how many classes are mixed in the two groups generated by the decision tree (27). We choose the number of leaves when the complexity parameter is the lowest to minimize the chance of making errors in the decision tree.

### Logistic Regression

In the training dataset, we used the Least Absolute Shrinkage and Selection Operator (LASSO) method to select the most useful predictive variables (28). Continuous variables were made into dichotomous variables and were entered into a logistic regression with other variables. The nomogram predicting the occurrence of AKD was established using the LASSO method for the selected variables. The performance of the nomogram was evaluated by calibration curves. The calibration evaluation uses a calibration chart to show the relationship between the observed frequency and the predicted probability. The nomogram was verified in the validation dataset to evaluate the stability of the nomogram. In addition, decision curve analysis (DCA) was used to evaluate the clinical utility of the final nomogram (29). The net benefit is calculated by subtracting the proportion of false positives from true positives (30).

Moreover, the discrimination of three machine learning algorithms in predicting the occurrence of AKD patients was compared using Delong's method. The discrimination was validated externally by the AUROC in the MIMIC III database.

We performed all statistical analyzes using R software version 4.0.5 (R Foundation for Statistical Computing).

## Results

### Participants

**Figure 1:**
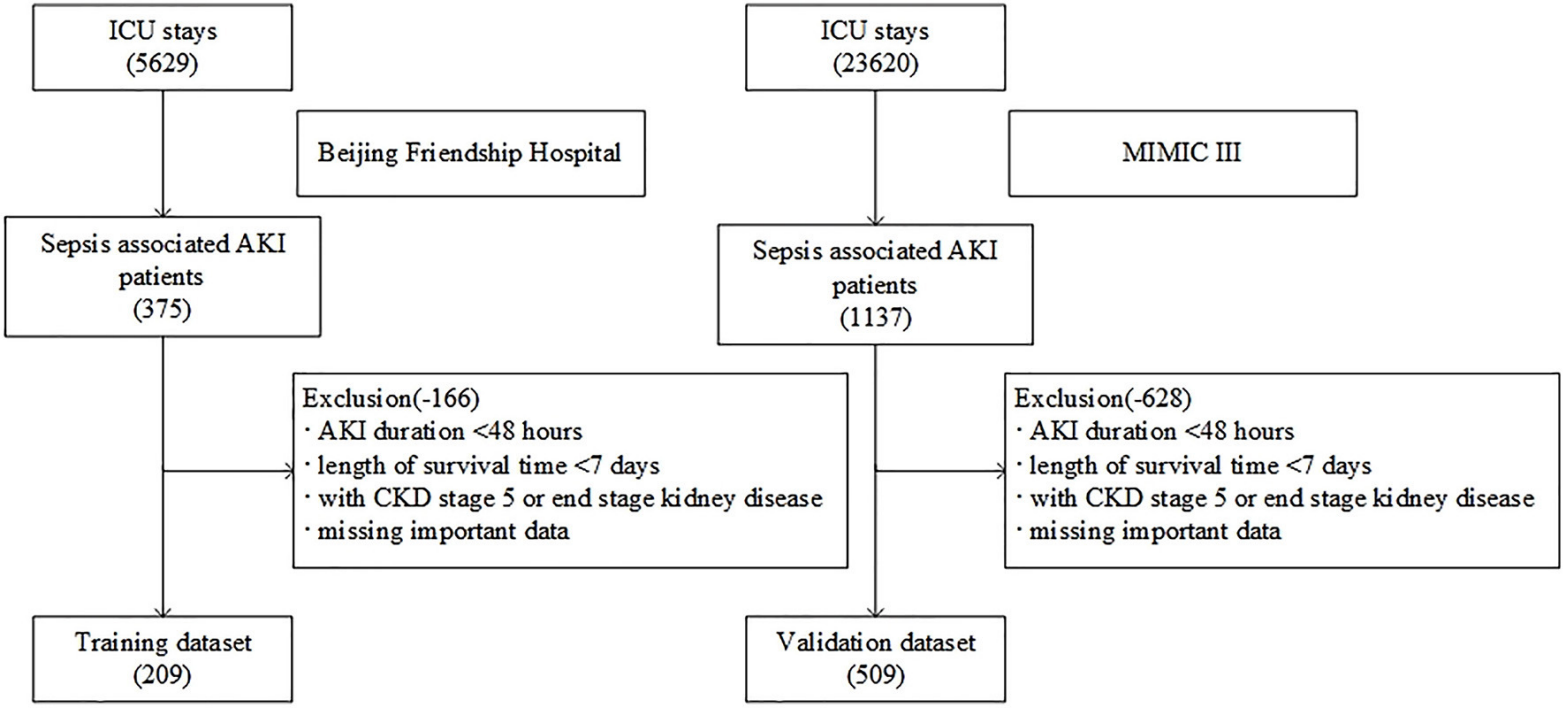
Flow chart of patient selection.

As shown in Figure 1, a total of 5,629 patients were screened during the study period in the BFH. The initial research identified 23,620 ICU admissions from the MIMIC III database. In addition, 209 and 509 patients were assigned to the training dataset and validation dataset, respectively. Twenty-eight predictors were extracted from the database and included in the model. The occurrence of AKD rate was 55.5% (116 patients with AKD) in the training dataset and 46.4% (236 patients with AKD) in the validation dataset. A comparison of baseline characteristics between the AKD group and non-AKD group in BFH and MIMIC-III cohorts are recorded in Table 1. AKD patients were older and had higher Charlson score and delta non-renal SOFA; higher creatinine at day 3 and AKI stage; more medical history of hypertension, diabetes mellitus, and CKD; more application of diuretics and renal toxic drugs in the training dataset (*p* < 0.05), while they had a lower delta creatinine, urine output at day 3, and delta urine output (*p* < 0.05). Furthermore, comorbidities of CKD, higher AKI stage, and lower delta creatinine also showed similar results between AKD patients and non-AKD patients in the validation dataset (*p* < 0.05). Our study was reported according to the guidelines of the TRIPOD statement.

**Table 1:** Baseline characteristics of the Beijing Friendship Hospital (BFH) and Medical Information Mart for Intensive Care III (MIMIC III) cohorts.

	**BFH cohort**			**MIMIC III cohort**		
	**Non-AKD (*n* = 93)**	**AKD (*n* = 116)**	***P*-value**	**Non-AKD (*n* = 273)**	**AKD (*n* = 236)**	***P*-value**
Age, mean (SD)	54.7 (20.7)	64.5 (14.7)	<0.001	64.3 (16.5)	62.6 (18.1)	0.252
Male, (%)	58 (62.4)	74 (63.8)	0.832	160 (58.6)	117 (49.6)	0.041
BMI, kg/m^2^, median [Q1, Q3]	26.6 (22.5, 30.4)	26.3 (22.4, 29.4)	0.837	27.3 (23.5, 32.2)	27.3 (23.3, 31.2)	0.782
Heart failure, *n* (%)	19 (20.4)	30 (25.9)	0.357	66 (24.2)	50 (21.2)	0.423
Hypertension, *n* (%)	45 (48.4)	74 (63.8)	0.025	21 (7.7)	30 (12.7)	0.060
Chronic obstructive pulmonary disease, *n* (%)	12 (12.9)	19 (16.4)	0.482	62 (22.7)	61 (25.8)	0.410
Chronic liver disease, *n* (%)	3 (3.2)	6 (5.2)	0.491	30 (11.0)	27 (11.4)	0.872
Diabetes mellitus, *n* (%)	35 (37.6)	66 (56.9)	0.006	76 (27.8)	52 (22.0)	0.132
Chronic kidney disease, *n* (%)	37 (39.8)	26 (22.4)	0.007	36 (13.2)	18 (7.6)	0.042
Charlson score, median [Q1, Q3]	2 (1, 3)	2 (1, 4)	0.001	2 (1, 3)	2 (1, 3)	0.729
Emergency department, *n* (%)	59 (63.4)	75 (64.7)	0.856	33 (12.1)	36 (15.3)	0.298
Surgery, *n* (%)	25 (26.9)	20 (17.2)	0.092	99 (36.3)	56 (23.7)	0.002
APS III, median [Q1, Q3]	45 (32, 62)	44.5 (32, 63)	0.779	40 (29, 55)	36.5 (26, 48)	0.035
SAPS II, median [Q1, Q3]	35 (25, 46)	35 (27, 44)	0.779	31 (23, 44)	31.5 (23, 41)	0.461
Non-renal SOFA at day 1, median [Q1, Q3]	3 (1, 6)	3 (1, 6)	0.310	3 (1, 5)	2 (1, 4)	0.044
Non-renal SOFA at day 3, median [Q1, Q3]	3 (1, 6)	3 (1, 6)	0.375	2 (1, 4)	2 (1, 4)	0.226
Delta non-renal SOFA, median [Q1, Q3]	0 (0, 0)	0 (0, 1)	<0.001	0 (0, 0)	0 (0, 0)	0.478
AKI stage, *n* (%)			<0.001			0.008
1	13 (14.0)	3 (2.6)		132 (48.4)	145 (61.4)	
2	33 (35.5)	20 (17.2)		91 (33.3)	55 (23.3)	
3	47 (50.5)	93 (80.2)		50 (18.3)	36 (15.3)	
Baseline creatinine, mg/dl, median [Q1, Q3]	0.7 (0.5,1.0)	0.8 (0.5, 1.1)	0.880	0.6 (0.5, 0.9)	0.60 (0.4, 0.9)	0.700
Creatinine at day 1, mg/dl, median [Q1, Q3]	1.3 (0.9, 2.2)	1.4 (0.9, 2.5)	0.857	1.1 (0.9, 1.5)	1.0 (0.8, 1.4)	0.050
Creatinine at day 3, mg/dl, median [Q1, Q3]	1.1 (0.9, 1.6)	1.2 (0.8, 2.0)	0.014	1.0 (0.8, 1.3)	0.9 (0.7, 1.4)	0.159
Delta creatinine, mg/dl, median [Q1, Q3]	−0.1 (−0.7, 0.0)	−0.1 (−0.40, 0.0)	0.001	−0.1 (−0.20, 0.0)	0.0 (−0.1, 0.0)	<0.001
Urine output at day1, ml/kg/h, median [Q1, Q3]	0.9 (0.4, 2.9)	0.9 (0.4, 2.8)	0.457	1.9 (0.7, 3.9)	1.9 (0.8, 4.0)	0.324
Urine output at day3, ml/kg/h, median [Q1, Q3]	1.1 (0.7, 1.7)	0.9 (0.3, 1.5)	<0.001	1.0 (0.6, 1.6)	1.1 (0.7, 2.1)	0.438
Delta urine output, ml/kg/h, median [Q1, Q3]	1.1 (0.7, 1.7)	0.9 (0.3, 1.5)	<0.001	−0.3 (−1.7, 0.0)	−0.2 (−1.0, 0.0)	0.219
Diuretics, *n* (%)	22 (23.7)	94 (81.0)	<0.001	126 (46.2)	91 (38.6)	0.084
Mechanical ventilation, *n* (%)	61 (65.6)	74 (63.8)	0.787	137 (50.2)	103 (43.6)	0.141
Renal toxic drugs, *n* (%)	46 (49.5)	89 (76.7)	<0.001	115 (42.1)	84 (35.6)	0.132

*MIMIC III, Medical Information Mart for Intensive Care III; AKD, acute kidney disease; BMI, body mass index; APS III, Acute Physiological Score III; SPAS II, Simplified Acute Physiology Score II; SOFA, Sequential Organ Failure Assessment*.

### Model Development

**Figure 2:**
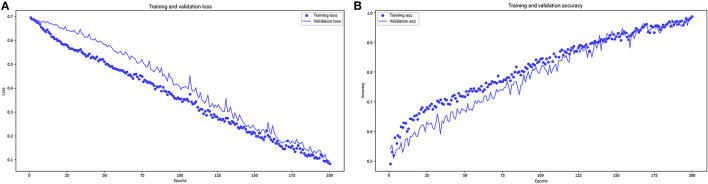
Loss **(A)** and accuracy **(B)** vs. epoch graph (up to 200 epochs).

**Figure 3:**
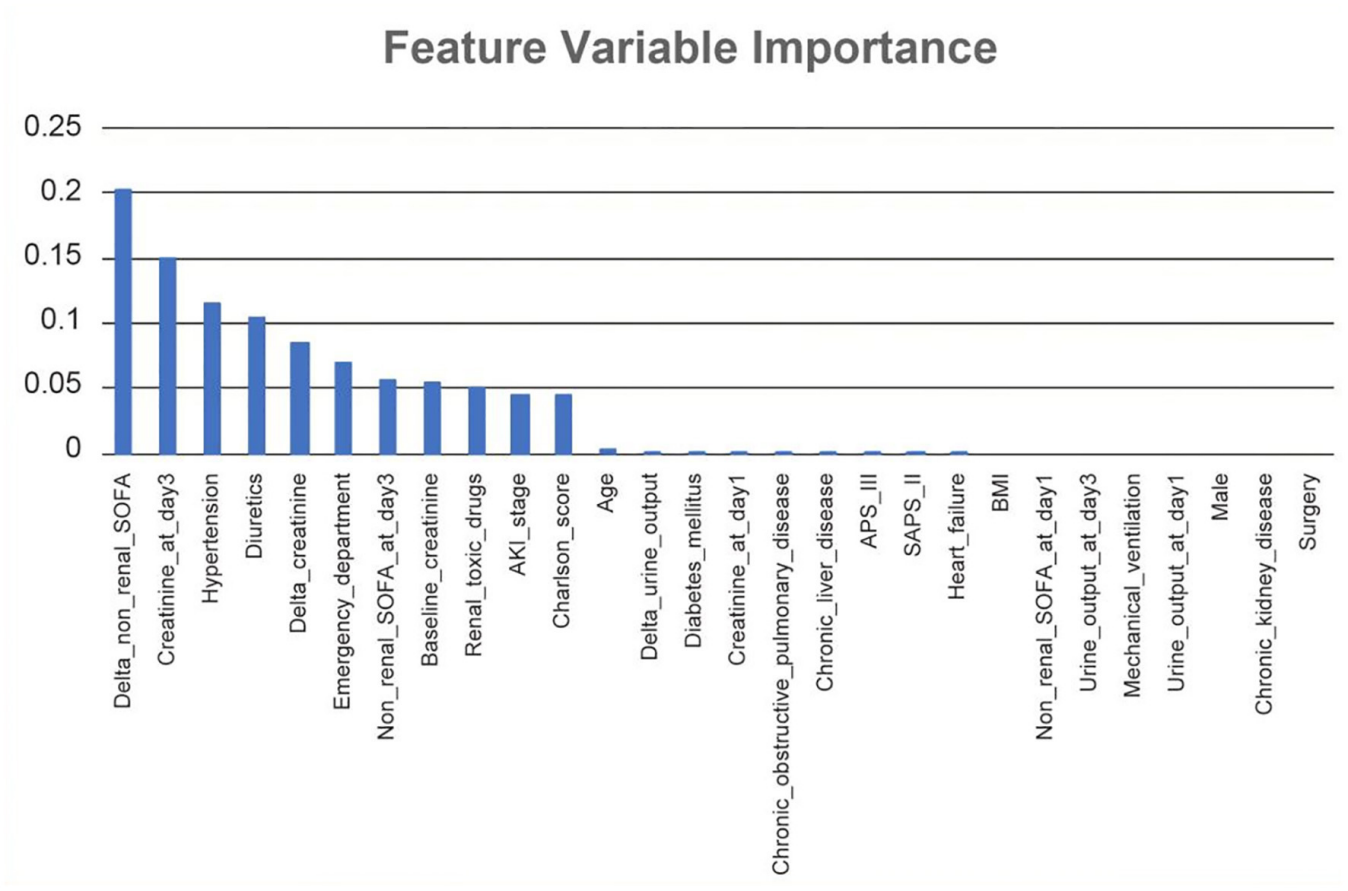
Significance of the predictors in the Recurrent Neural Network-Long Short-Term Memory (RNN-LSTM) model. All 28 important features regarding the development of the final predictive model are depicted.

**Figure 4:**
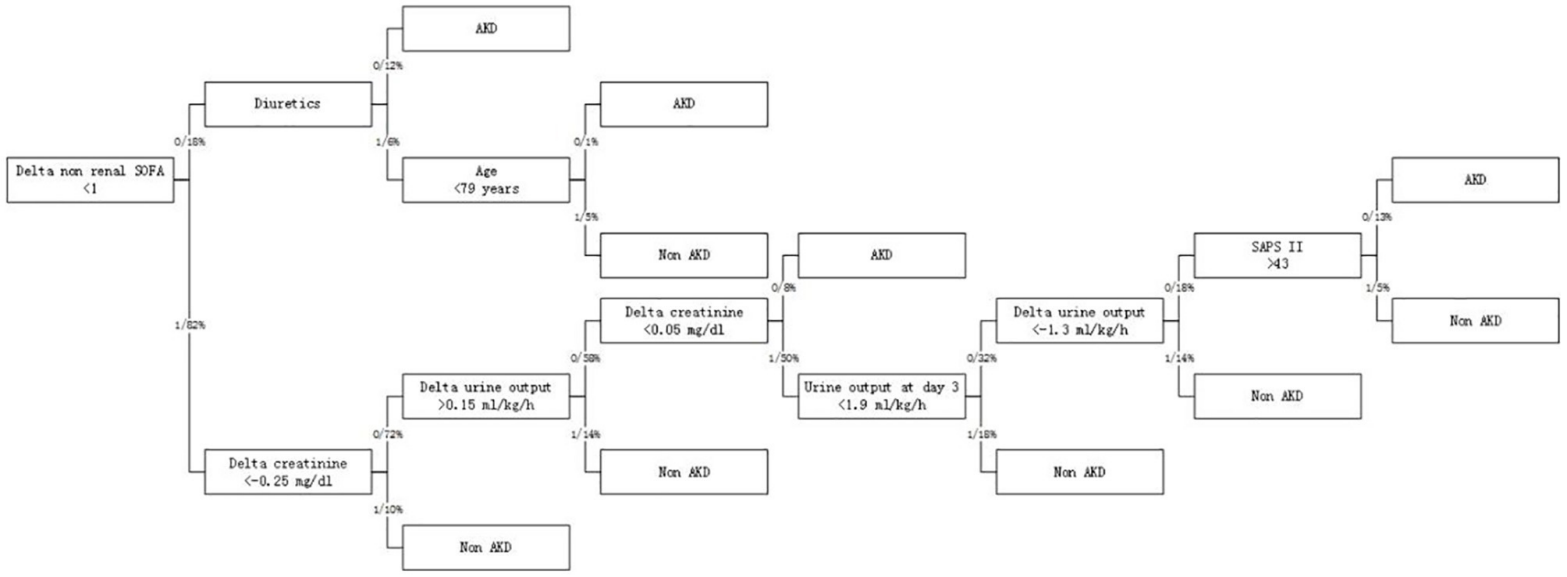
Contribution of 28 variables in predicting the occurrence of patients with sepsis-associated AKD.

**Figure 5:**
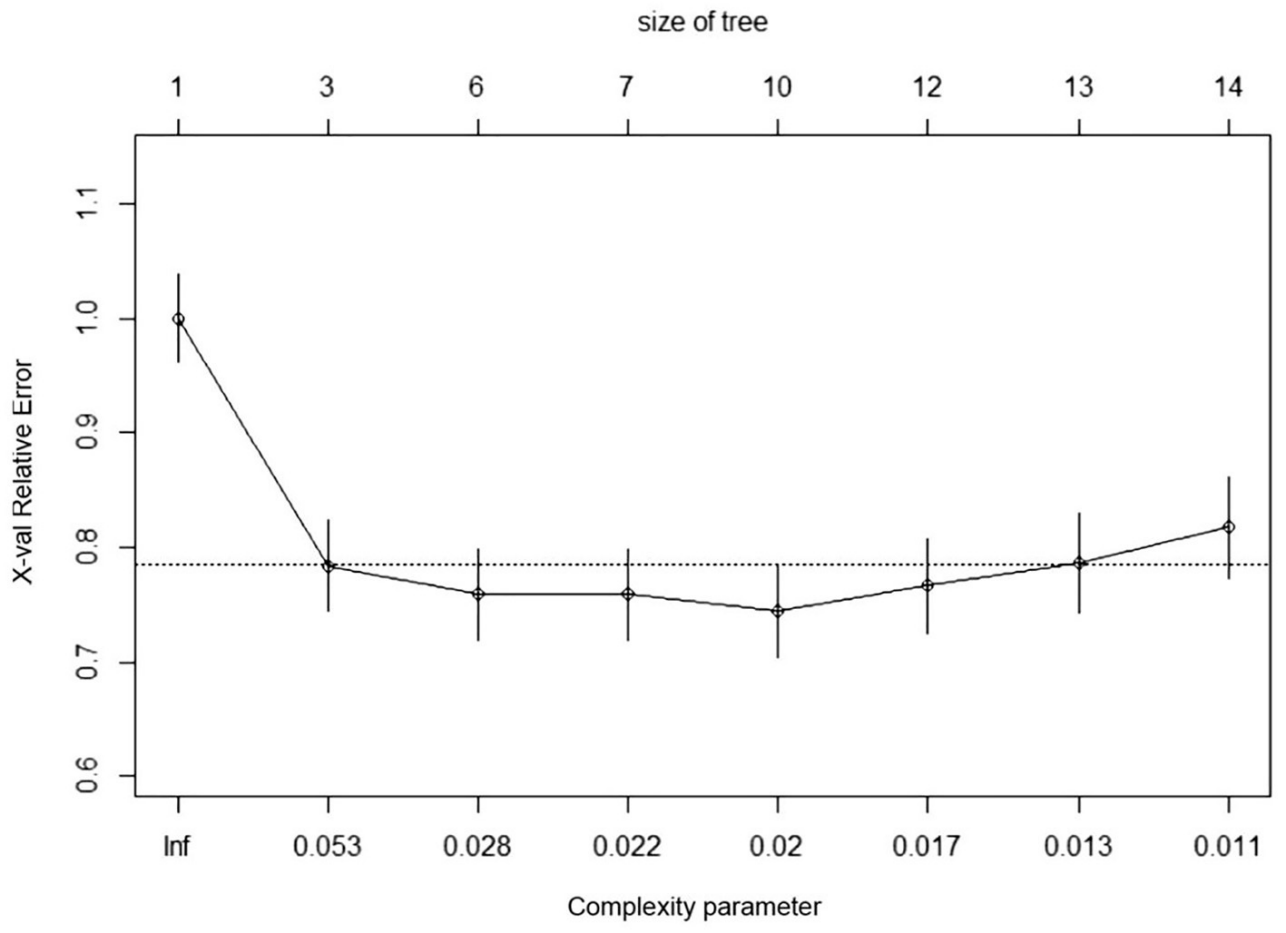
Optimized decision tree for the classification of acute kidney disease (AKD)/non-AKD of patients.

In RNN-LSTM, as the validation loss was decreasing over time, the accuracy of the model increased (Figure 2). The LSTM has been trained up to 200 epochs to obtain the smallest loss and the greatest accuracy. Throughout the training process of 200 epochs, our training loss and validation loss had decreased and accuracy increased gradually, respectively. At the 200th epoch, the training loss and the validation loss are approximately the lowest, where the training accuracy and the validation accuracy reach 97.96 and 97.66%, respectively. We found that the training graph and the validation graph are quite similar. Thus, it can be concluded that the model is quite accurate. It is neither overfitting nor underfitting. The significance of the predictors in the RNN-LSTM model is presented in Figure 3. The feature variable importance showed that Δnon-renal SOFA had an important role. Other variables, such as creatinine on day 3, hypertension, and diuretics, also showed marked effects. As the decision trees algorithm has nodes that represent variables and conjunction that connects the nodes, the performance of this algorithm mainly depends on the number of nodes and tree size (31). We explored different ways to find the optimal performance of the decision trees algorithm by adjusting the number of nodes (Figure 4). We found that the optimal number of nodes that could minimize the decision trees' misclassification error rate was 10, where the complexity parameter was 0.018. Using this number of nodes, the decision trees' structure was pruned. Among these variables, Δnon-renal SOFA had a crucial role in the prediction of the occurrence of AKD. If Δnon-renal SOFA < 1, delta creatinine played an important role in the next decision. If Δnon-renal SOFA > 1, whether used diuretics or not was important. If Δnon-renal SOFA > 1 and patients did not receive diuretics, he/she was more likely to be diagnosed with AKD soon (Figure 5).

**Figure 6:**
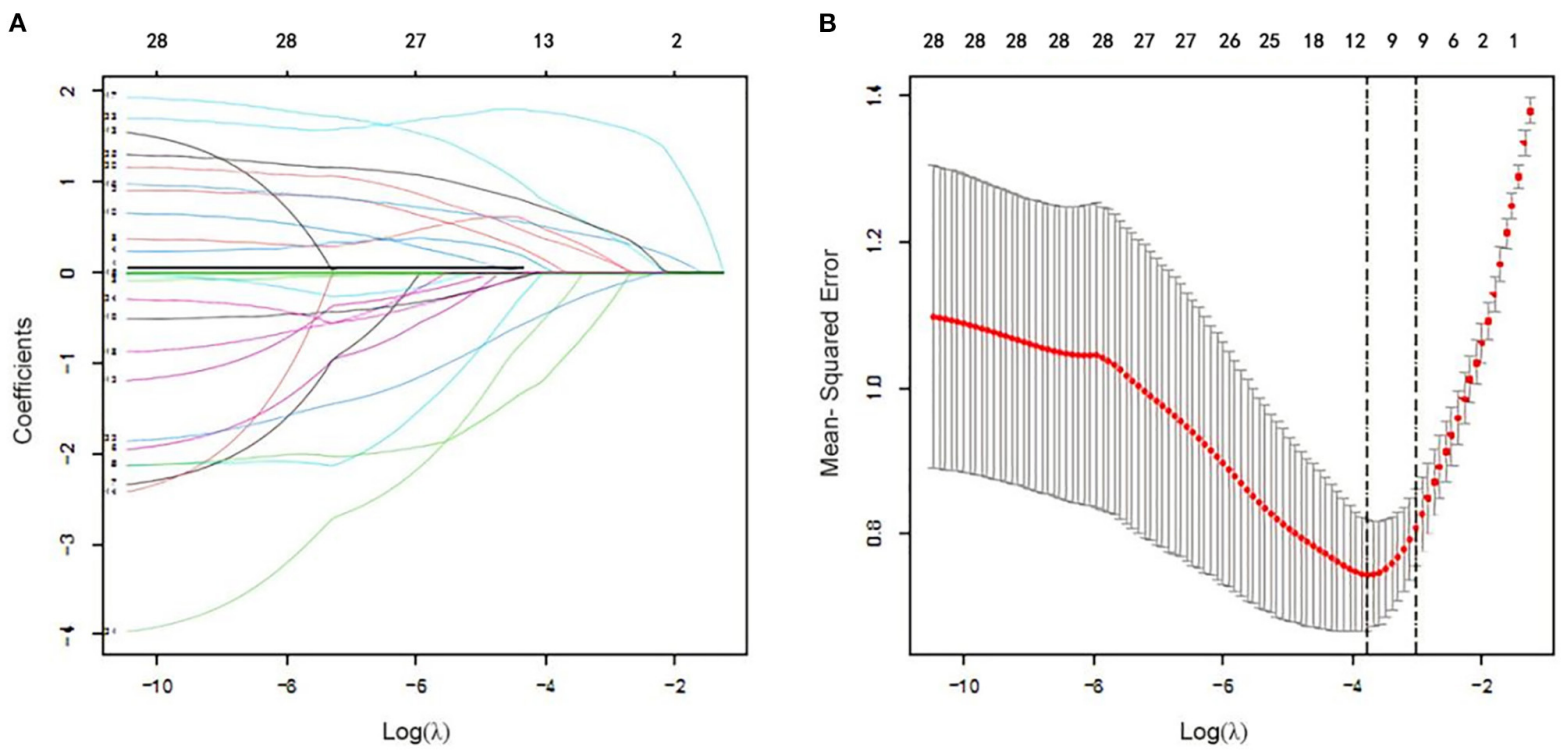
Clinical feature selection using the Least Absolute Shrinkage and Selection Operator (LASSO) logistic regression. **(A)** Optimal parameter (lambda) selection in the LASSO logistic regression. The black vertical lines were drawn at the optimal values by using the minimum criteria and the one SE of the minimum criteria (the 1-SE criteria). **(B)** LASSO coefficient profiles of the 28 features. A coefficient profile plot was produced against the log (lambda) sequence.

**Figure 7:**
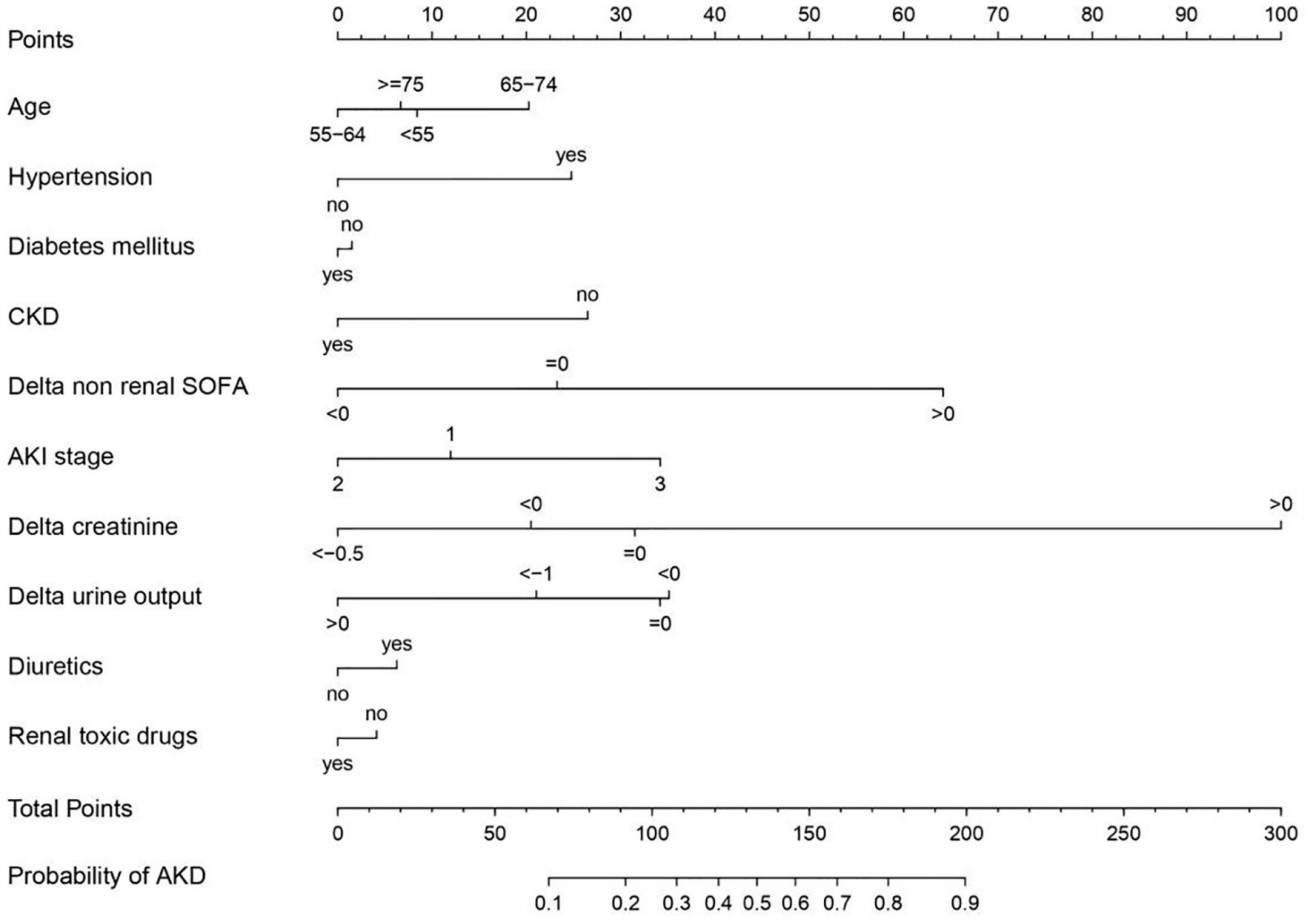
Nomogram developed based on the training dataset with the incorporation of age, combined with hypertension, diabetes mellitus, chronic kidney disease (CKD), delta non-renal Sequential Organ Failure Assessment (SOFA), acute kidney injury (AKI) stage, delta creatinine, delta urine output, diuretics, and renal toxic drugs.

**Figure 8:**
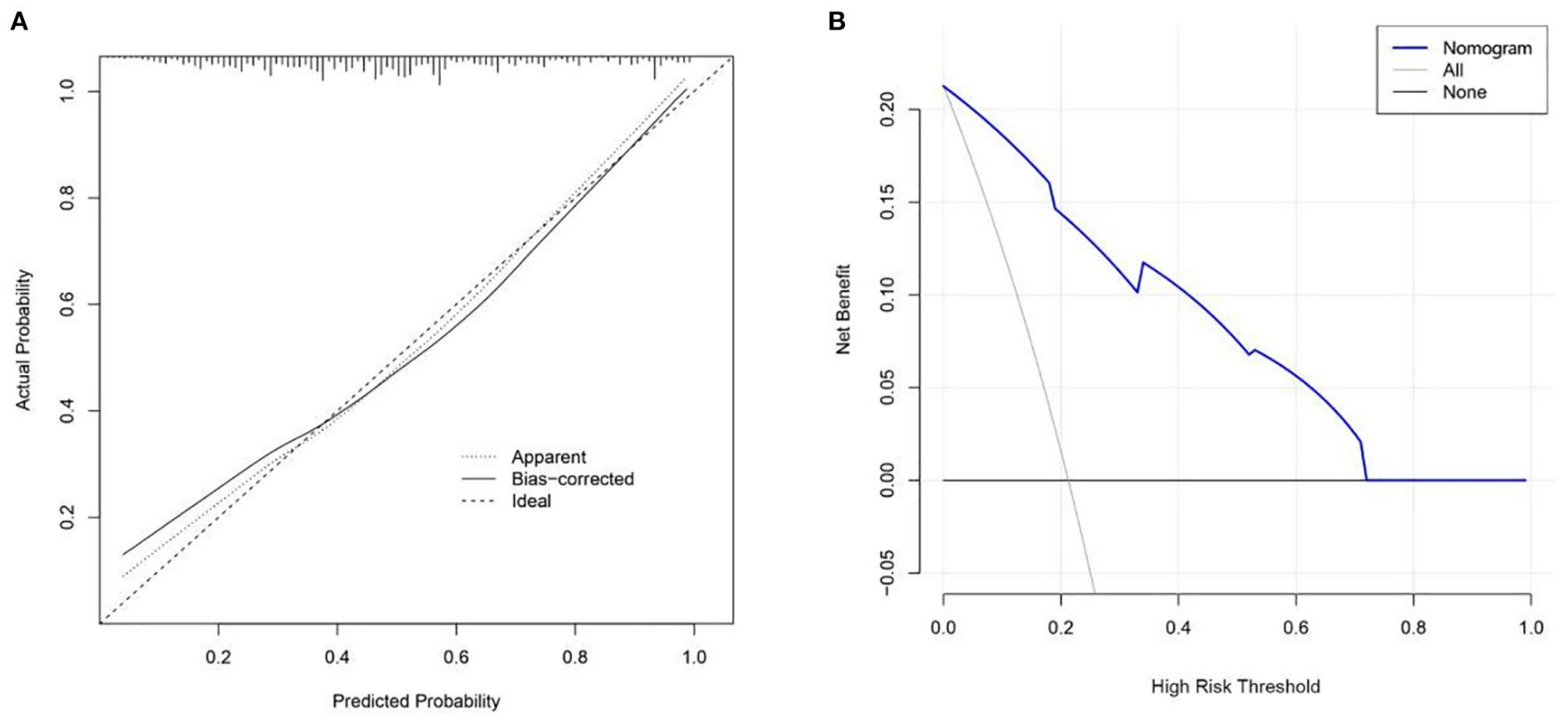
Calibration curves **(A)** and decision curve analysis **(B)** for nomogram.

In logistic regression, twenty-eight variables were included in the LASSO regression analysis and narrowed down to 10 features in the LASSO regression model (Figure 6). Next, a model integrating age, combined with hypertension, diabetes mellitus, CKD, delta non-renal SOFA, AKI stage, delta creatinine, delta urine output, diuretics, and nephrotoxic drugs was established using the training dataset. Based on this model, a nomogram was plotted to predict the probability of the occurrence of AKD patients (Figure 7). The calibration curve was described using the bootstrap method for both, the training and validation datasets (Figure 8A). The apparent line and a bias-corrected line only slightly deviated from the ideal line, indicating a good agreement between the prediction and reality. The DCA curve was plotted to perform a clinical application of this nomogram. In the training dataset, clinical intervention guided by this nomogram provided a greater net benefit when the threshold probability was within 0.01 and 0.71 (Figure 8B).

### Model Performance

**Figure 9:**
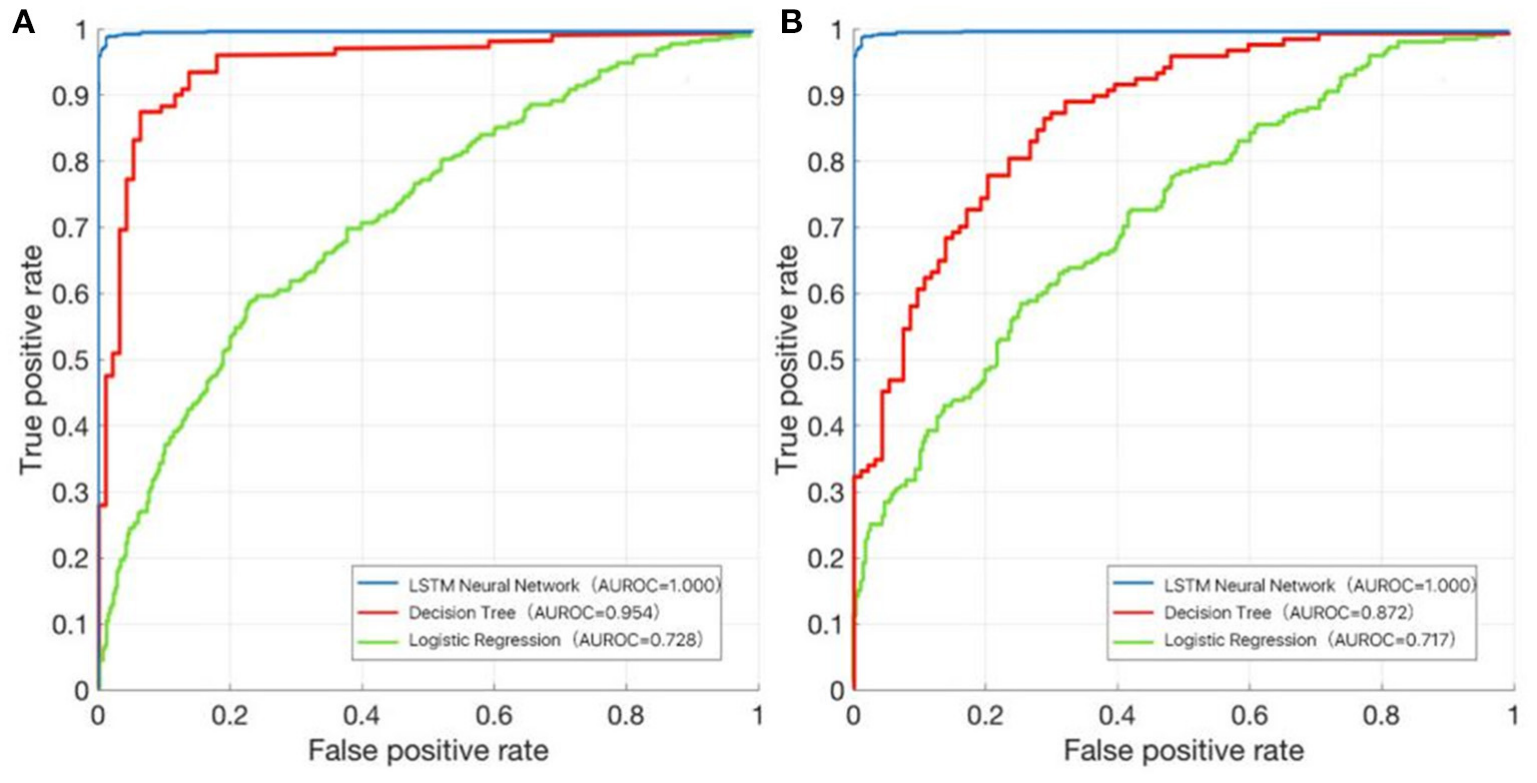
The area under the receiver operating characteristic (AUROC) curve of the RNN-LSTM, decision trees, and logistic regression. **(A)** Training dataset; **(B)** Validation dataset.

In the training dataset, we evaluated the discrimination of three models. RNN-LSTM was well-discriminated in the external validation dataset (AUROC: 1), which was greater than decision trees and logistic regression (AUROC: decision trees 0.954, logistic regression 0.728; Figure 9A). In the validation dataset, among RNN-LSTM, decision trees, and logistic regression algorithms, the RNN-LSTM algorithm showed the highest performance with an AUROC of 1.000, followed by the decision trees with an AUROC of 0.872. Logistic regression had the least predictive accuracy, with an AUROC of 0.717. All machine learning models, except the logistic regression model, showed good discrimination ability in the training and validation datasets. In the training and validation datasets, the RNN-LSTM algorithm achieved the best performance among the four models (Figure 9B).

## Discussion

In the present study, a total of 209 patients from BFH were included, with 55.5% of them diagnosed as having AKD. Using the data from BFH and MIMIC III records, we successfully developed and validated machine learning models to predict the occurrence of AKD in patients with AKI.

Since the diagnostic criteria for AKD were released in ADQI-16, several investigations have been undertaken on the epidemiology of AKD. Kellum et al. reported the incidence rate of AKD as 36.2% in ICU patients (4). Federspiel et al. showed the incidence rate of sepsis-associated AKD as 32.4% in critically ill patients (5). Peerapornratana et al. reported the incidence rate of sepsis-associated AKD in patients dying within 7 days was 33.6% (161/479) from the first day of being diagnosed with AKI (11). Our studies showed an AKD diagnosis rate of 55.5%. This higher rate could be attributed to the exclusion of patients with an AKI duration of <3 days.

Ostermann et al. suggested that nephrotoxic drugs increase the risk of renal function impairment (32). Drugs are among the main causes of AKI. Its pathogenesis included acute tubular necrosis, tubular obstruction by crystals or casts, and interstitial nephritis induced by drugs and their metabolites (33). Our study shows that nephrotoxic drugs increase the incidence of AKD, possibly because they deteriorate renal function.

There has been a controversy about whether the application of diuretics can improve renal function in recent years. A Phase II Randomized Blinded Controlled Trial of the Effect of furoSemide in Critically Ill Patients With eARly Acute Kidney Injury (SPARK-RCT) study showed that diuretics improved neither the recovery rate of AKI nor the prognosis of the patients (34). The study of Zhao et al. reported that administering diuretics improved renal function in patients on the MIMIC III database (35). Our research shows that the use of diuretics may be related to the low incidence of AKD. The effective use of diuretics can reflect the recovery of the patients' renal function, but it may not change it. More research is needed to further clarify the role of diuretics in improving renal function.

There are some studies on the prediction of AKD in hospitalized patients with AKI. Zhao et al. used multivariable logistic regression analysis with the LASSO method to select features and build a nomogram (36). The model displayed good predictive power with an AUROC of 0.834 (95% CI:0.773–0.895) in the training dataset and an AUROC of 0.851 (95% CI:0.753–0949) in the validation dataset. Yan et al. also established a prediction model using multivariable logistic regression analysis (37). The 8-variable model showed good discrimination and calibration in predicting AKD stage 2–3 with the AUROC being 0.85 (95% CI:0.83–0.87). Xiao et al. established a prediction model using multivariable logistic regression analysis. This model showed a large AUROC (0.879 ± 0.009, 0.879 ± 0.011) and had stable sensitivity (81 and 82%) and specificity (81 and 80%) in derivation cohort and validation dataset, respectively (38). In our study, the AUROCs of the logistic regression model were 0.728 (training dataset) and 0.717 (validation dataset), which were lower than the above studies. This may be due to differences in the study population. A study by Tuan et al. studied sepsis-associated AKI patients, however, they predicted progression to chronic kidney disease rather than AKD (39). Therefore, to our knowledge, this is the first study to use longitudinal data to predict the occurrence of AKD with the application of machine learning.

To identify AKD patients, an important strength of our study was the use of new criteria of sepsis-associated AKI, and this method would overcome some inherent weaknesses of using hospital discharge data (40, 41). The delta non-renal SOFA contains only 5 simple variables recorded in clinical routines. Therefore, if implemented, the delta non-renal SOFA will not require manual input of additional variables as the model is based on variables routinely collected. In our study, for predicting the occurrence of AKD, the delta non-renal SOFA score had high discriminatory power. The delta non-renal SOFA is simple for calculation and easy to use and has robust discrimination and calibration. To predict the occurrence of AKD patients with sepsis, ICU physicians could use the delta non-renal SOFA and improve clinical decision-making at the bedside. Moreover, the predictor variables that we used were quite universally obtained in the emergency department. After further validation and recalibration, the delta non-renal SOFA appeared to have the potential to help emergency department clinicians triage decisions and ICU placement.

### Limitations

The study has the following limitations. First, we chose to analyze the patients admitted to the ICU with sepsis. There were certainly patients who had been diagnosed with sepsis before or after the ICU admission, but we limited our study population to those who fulfilled sepsis-3 criteria during their 1st day in ICU. Second, we have a limited number of patients and a small sample size, but we conducted an external validation by using the data of 509 sepsis-associated AKI patients from the MIMIC III database, and the results indicated that the calibration of delta non-renal SOFA was relatively well with accordance of occurrence of AKD. Finally, we prepared our dataset from the retrospective database, and the outcomes of sepsis-associated AKI patients could have changed over time due to the update of treatment guidelines and advances in treatment and diagnostic technology.

## Conclusion

Machine learning could be applied to the predictive AKD, and it is where the RNN-LSTM model works the best. The non-renal SOFA plays an important role in predicting the AKD.

## Data Availability Statement

The raw data supporting the conclusions of this article will be made available by the authors, without undue reservation.

## Ethics Statement

The studies involving human participants were reviewed and approved by institutional review boards of Beijing Friendship Hospital and the Massachudatasetts Institute of Technology. Written informed consent for participation was not required for this study in accordance with the national legislation and the institutional requirements.

## Author Contributions

JH and MD conceived the idea, performed the analysis, and drafted the manuscript. JH and JL interpreted the results and helped to revise the manuscript. JL and MD helped to frame the idea of the study and helped to analyze the data. All authors read and approved the final manuscript.

## Funding

This work was supported in part by grants from the Beijing Key Clinical Specialty Excellence Project.

## Conflict of Interest

The authors declare that the research was conducted in the absence of any commercial or financial relationships that could be construed as a potential conflict of interest.

## Publisher's Note

All claims expressed in this article are solely those of the authors and do not necessarily represent those of their affiliated organizations, or those of the publisher, the editors and the reviewers. Any product that may be evaluated in this article, or claim that may be made by its manufacturer, is not guaranteed or endorsed by the publisher.
